# Computer vision mechanical QA: Development, characterization, and five years of clinical performance

**DOI:** 10.1002/acm2.70678

**Published:** 2026-07-15

**Authors:** Rachel B. Ger, Michael D. Armstrong, Daniel G. Robertson

**Affiliations:** ^1^ Department of Radiation Oncology Mass General Brigham Cancer Institute Boston Massachusetts USA; ^2^ Healthcare Technology Management Mayo Clinic Phoenix Arizona USA; ^3^ Department of Radiation Oncology Mayo Clinic Phoenix Arizona USA

**Keywords:** computer vision, mechanical QA, quality assurance

## Abstract

**Background:**

Quality assurance (QA) in radiation therapy is a critical component of ensuring accurate and consistent patient treatments, but many tasks are time consuming and rely on human visual acuity, which limits their accuracy.

**Purpose:**

We aimed to automate monthly mechanical QA tests, improve measurement accuracy and precision, and decrease inter‐user variability using a computer vision‐based quality assurance (CVQA) system.

**Methods:**

A custom marker board incorporating four ArUco markers was created along with a custom camera holder that mounted onto the gantry head. OpenCV was utilized to automate tests for couch translation, collimator and table angles, collimator and table walkout, optical distance indicator (ODI), and field size detection. A GUI was created to guide users through the tests, determine passing status of measurements based on MPPG8.b criteria, and display image captures to allow users to troubleshoot if needed. Reproducibility tests were taken across four days. The system was tested against manual measurements by graph paper or digital level for each test. Field sizes and ODI were read by 12 different physicists to determine human variability and comparisons for CVQA.

**Results:**

The CVQA system took 5 min to set up and 7 min to perform all tests. All field sizes, collimator/table angles, collimator/table walkout, and table translations were reproducible within 0.5 mm and 0.5°. ODI measurements were reproducible within 1 mm. Table travel, ODI, and walkout measurements agreed with manual measurements within 0.5 mm and 0.4°, except for vertical table motions that agreed within 0.9 mm due to the lens focus being optimized for the 100 cm SSD plane. The standard deviation between physicists for almost all symmetric and asymmetric jaws was larger than the reproducibility of CVQA. CVQA has been utilized for five years and has demonstrated ability to identify mechanical machine issues.

**Conclusions:**

CVQA successfully automates mechanical QA tasks, providing an efficient and precise system. CVQA is open source and freely available for academic institutions. Its adoption can improve workflow efficiency and consistency in clinical environments.

## 1 INTRODUCTION

Quality assurance (QA) in radiation therapy is a critical component of ensuring accurate and consistent patient treatments. Modern linear accelerators (linacs) require extensive monthly mechanical QA tests as outlined by the American Association of Physicists in Medicine (AAPM) Task Group 142 (TG‐142) and updated by Medical Physics Practice Guideline 8.b (MPPG 8.b).^1^, ^2^As the tests have action limits in millimeters, the accuracy should be sub‐millimeter in order to determine properly if the test is exceeded. Traditionally, these tests rely on the subjective visual assessment of physicists, which introduces inter‐ and intra‐user variability and limits reproducibility. The desired level of accuracy cannot always be maintained within sub‐millimeter by reliance on the human visual system. QA tools have been developed using digital sensors to measure gantry and collimator angle and ODI,^3^ but many mechanical tests still rely on human visual interpretation of features of the field light.

To overcome these limitations, researchers have explored automation using computer vision techniques. Jenkins et al. used a radio luminescent phantom with a gantry‐mounted camera and were able to automate light field and radiation coincidence, jaw position, cross‐hair walkout, couch lateral and longitudinal translations, and laser alignment.^4^ However, the phantom was relatively small so the largest field size used was 10 cm x 10 cm. Shandiz et al. also used a gantry‐mounted camera and a 3D phantom similar to Jenkins et al. and were able to automate optical distance indicator (ODI) and gantry angle.^5^ Kim et al. used a gantry‐mounted smartphone to automate detection of gantry angle, collimator angle, jaw position, crosshair walkout, and ODI.^6^ Unlike the others, no calibration pattern was used, instead a coin was used for scaling and internal cell phone sensors such as the gyroscope, compass, and accelerometer were used for analysis. Due to the camera field of view, jaw position detection was limited to field sizes of 30 cm x 30 cm or less. Park et al. differed from these previous studies and used a couch‐mounted camera to focus on radiation fields primarily.^7^ They were able to automate detection of linac isocenter, gantry rotation isocenter, collimator rotation isocenter, couch rotation isocenter, and couch position.

In this study, we developed Computer Vision QA (CVQA), a system designed to automate various mechanical QA tests, including couch translation (lateral, longitudinal, and vertical), collimator and table angles, collimator and table walkout, ODI, and field size detection. Our system features a graphical user interface (GUI) to facilitate clinical adoption with minimal training. Unlike previously published methods, CVQA does not require a separate calibration plate for each session use and allows measurements of all field sizes. To the best of our knowledge, this is the first device of its kind to be clinically utilized, which we report on our findings of utilizing CVQA clinically for 5 years.

## 2 METHODS

### 2.1 CVQA system design

**FIGURE 1:**
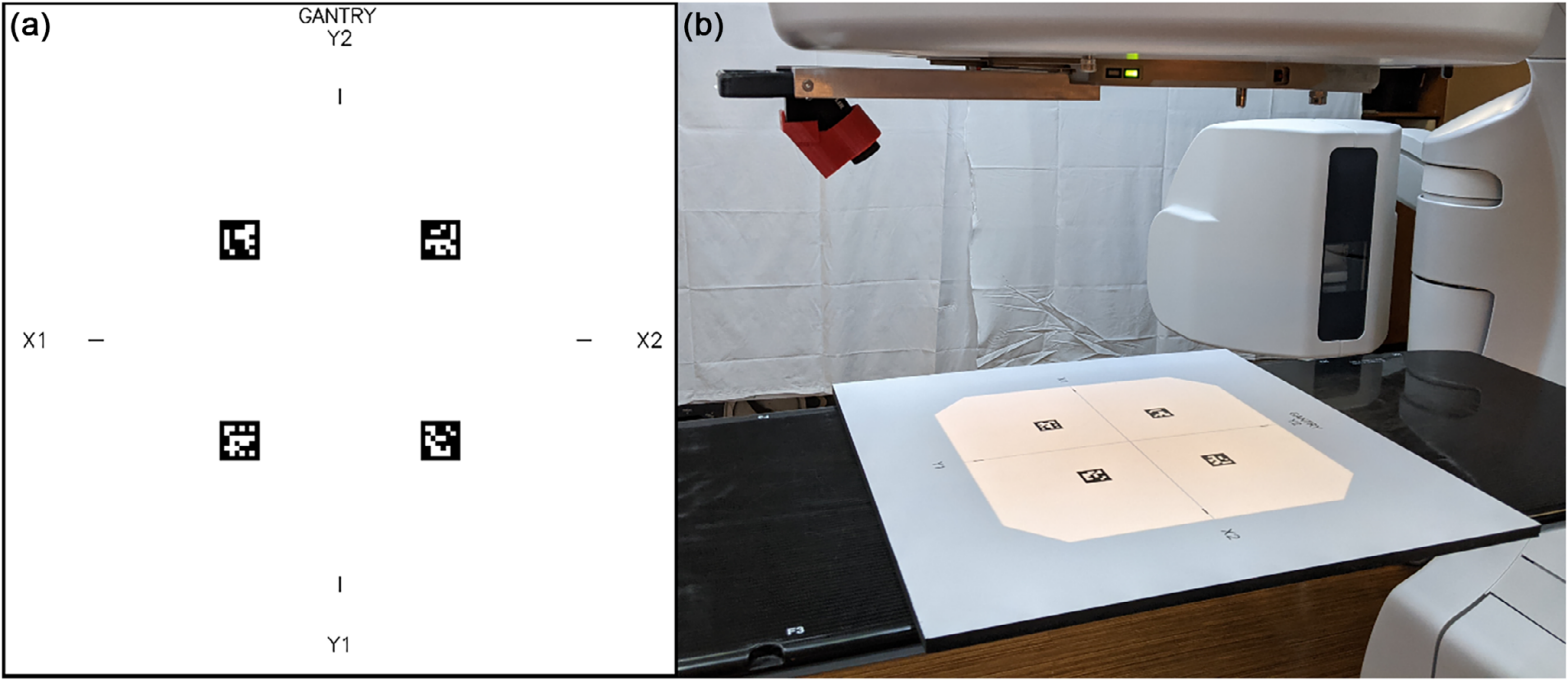
CVQA marker board and camera mount. (a) The marker board designed for the CVQA system, utilizing ArUco markers. (b) The CVQA camera mount attaches to the interface mount of a Varian TrueBeam. The CVQA marker board is shown on the table aligned to the crosshairs.

We designed a CVQA marker board that was custom manufactured (Calib.io ApS, Svendborg, Denmark). The CVQA board incorporates four ArUco markers, crosshair alignment marks, and references for the gantry and collimator orientations (Figure 1a). The marker board was designed so that the spacing of the four uniquely coded ArUco markers does not interfere with the fields being measured. The ArUco markers are 30 mm square with the outer corners of the four markers 180 mm apart and centered on the crosshair marks. ArUco markers have a black border with an inner binary matrix that is specific to a given identifier.^8^ These markers were chosen instead of the commonly used checkerboard pattern, as they can provide orientation information as well as on‐line camera calibration. Collimator and table angles at the cardinal angles would appear redundant if a standard checkerboard pattern was used. The CVQA cardinal angle measurements can be distinguished from each other due to the orientation information provided by the ArUco markers. The placement of the ArUco markers and alignment marks was chosen to avoid interference with the field light edges for the different field sizes measured.

A CVQA camera mount was developed to attach to the interface mount of a Varian TrueBeam (Varian Medical Systems, Palo Alto, CA), featuring an angled camera assembly optimized for capturing the entire 40 cm x 40 cm field of view at 100 cm source‐to‐surface distance (SSD) (Figure 1b). The camera mount and handle were 3D printed and attached to a TrueBeam interface mount tray via milled aluminum brackets. The system employs a Blackfly S GigE camera (FLIR Systems, Wilsonville, OR) with an 8.5 mm fixed focal length lens (model 58‐000, Edmund Optics, Barrington, New Jersey).

Camera calibration is required during commissioning and after any change has been made to the aperture or focus adjustments of the lens. Annual camera calibration is recommended. Accurate measurements rely on proper aperture and focus setup before camera calibration. Guidance on setting aperture and focus can be found in the linked repository. Calibration was performed using a Calib.io (Calib.io ApS, Svendborg, Denmark) 200 mm x 300 mm 9 × 14 ChArUco (OpenCV 3) (combination of a chessboard and ArUco) calibration board with 20 mm checkers, 15 mm markers, and ArUco dictionary DICT_5 × 5. A chessboard is a common OpenCV pattern. The ChArUco board has the advantage of more points than a conventional chessboard pattern and it is also robust to occluded and clipped images. Numerous images at different poses were acquired to ensure a good camera calibration.

We calibrated the camera while installed in the TrueBeam Interface Mount just as with normal CVQA operation. We developed a treatment plan to move the gantry, collimator, and couch to different positions to create thirty different calibration poses. We used a calibration utility based on the OpenCV ChArUco camera calibration example. The result was a calibration file that contains the camera matrix and distortion coefficients. The focal length estimates in the camera matrix were fine tuned to produce accurate measurements for the table height. This was done by using CVQA to measure field size and couch vertical offsets and adjusting the focal lengths using trial and error to match a precision setup established with a front pointer and graph paper. The focal length is adjusted until the CVQA measurement agrees with the front pointer within the test tolerance. This tuning is specific to the CVQA device and must be repeated each time the camera is recalibrated.

The image processing and computer vision measurement functions were implemented in C++ using the OpenCV library (version 4.1), which provided general image manipulation tools, computer vision‐specific functions such as camera calibration, and also access to ArUco functionality. Images are acquired using our application programming interface (API), which is based on Flir's Spinnaker (Teledyne FLIR, Wilsonville, OR) API. Our API has functions to convert and format the 2448 × 2048 pixel raw image from the Blackfly camera to an OpenCV Mat of the same resolution. Perspective transformed images have a resolution of 2000 × 2000 pixels which provides a scale of 0.25 mm per pixel. After acquisition, the image is processed using our API to apply the camera calibration and measure image features as described in section 2.2.

**FIGURE 2:**
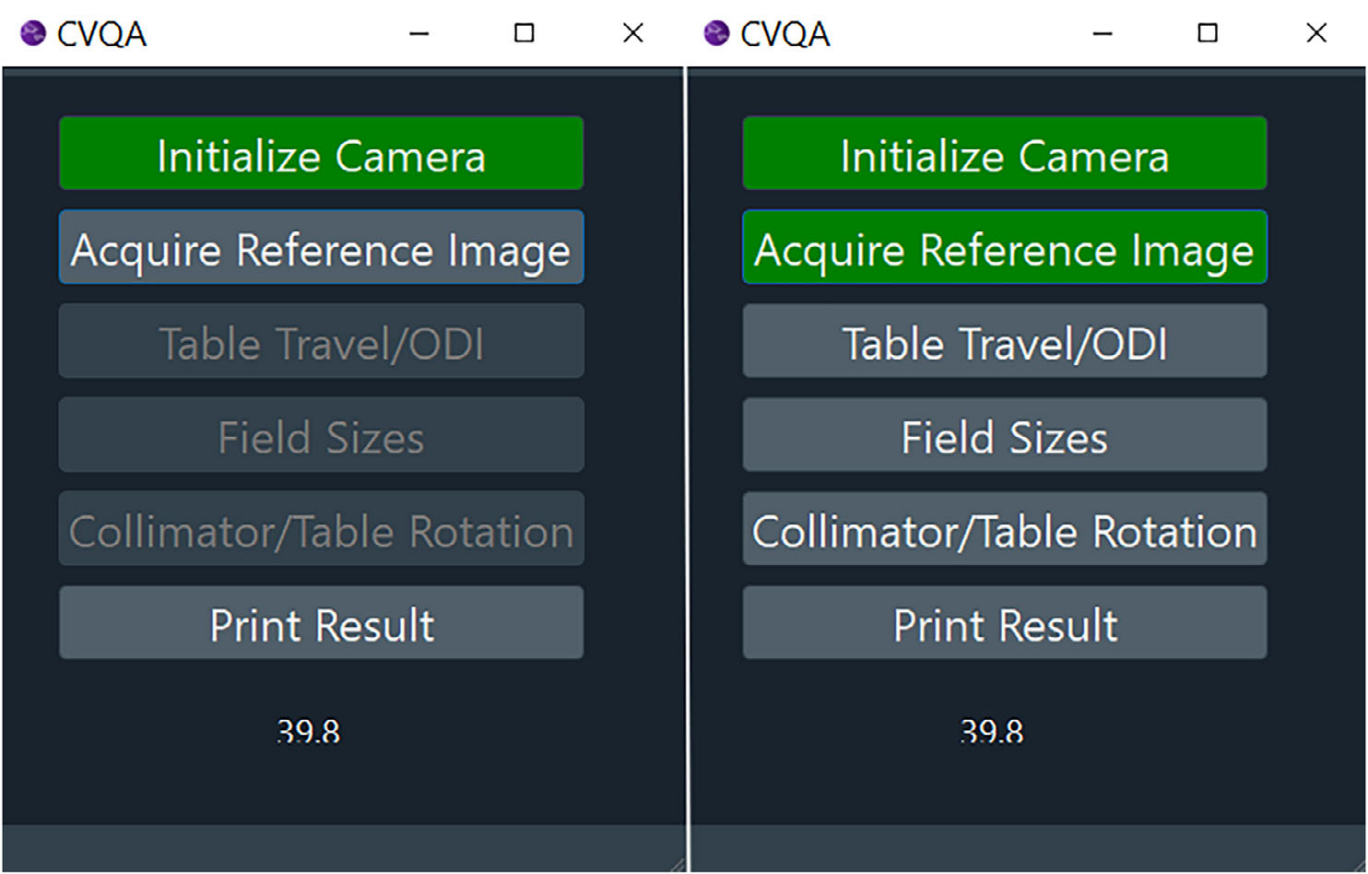
CVQA main GUI window. When first opening the software, users see the window on the left that forces them to perform the required preparation for QA tests along with the light level on the bottom to help guide adjustment of lights if needed. If camera initialization and reference image capture are completed successfully, the buttons turn green and the users can access all the mechanical QA tests in CVQA.

A GUI was created in C++ using Qt (Qt Creator version 4.12.4) that walks the user through necessary preparatory steps and then through each test. First, an initialization is performed that warms up the camera and checks that the ambient light is within the established bounds. The bottom of the CVQA menu displays the light level to aid in setup. Next, a reference image is acquired. The reference image uses the same processing steps to validate the marker board image and vertical offset as the other tests described below in the CVQA Mechanical Tests section. If either of these steps fail, the button turns red and the user is presented with a warning message describing the error. If both steps are successfully completed, then the buttons turn green and the user can press the buttons to open a window for each of the categories of mechanical QA tests. This is shown in Figure 2. For ease of use, a treatment plan was created with each mechanical test as a separate field. The user runs the plan in Dry Run Mode and steps through each field, which will drive the jaws, collimator, and table to the correct location for that test.

The CVQA code is available on GitHub at https://github.com/mikearmstrong800/computer_vision_mechanical_qa. The code is available under an academic use copyright license. The repository contains information for users to print their own CVQA board, ChArUco board, 3D printed handle and camera mount, and the GUI with all camera processing steps for the mechanical tests described.

### 2.2 CVQA mechanical tests

The mechanical QA tests that CVQA performs are grouped into three categories:

1. Table travel & ODI—Measures table vertical, longitudinal, and lateral translations as well as ODI verification.
2. Field sizes—Detects symmetric and asymmetric field sizes ranging from 5 cm × 5 to 40 cm × 40 cm, including jaw over‐travel measurements.
3. Collimator & table rotation—Measures collimator and table angles along with their respective walkout assessments.

**FIGURE 3:**
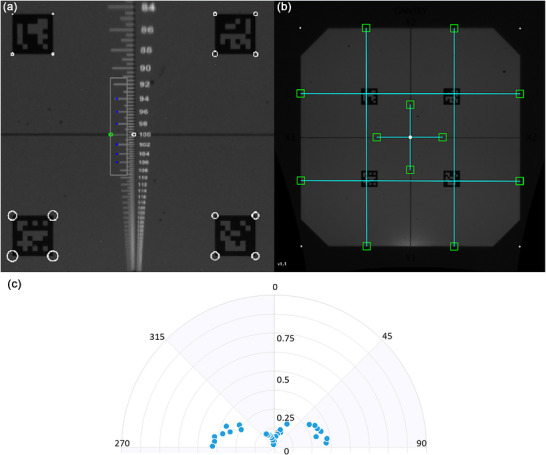
CVQA GUI images for user assessment. (a) The image seen by the user for ODI measurements. A box is automatically placed around the tick marks near the ODI, with blue circles identifying the location of the center of each tick mark. The crosshair detection is also shown with a green circle, and the field center is indicated by a white circle. The user can check that each of these locations was detected correctly. (b) The image users would see on field sizes. There are two boxes located along each field edge. Lines connect from boxes opposite one another. The end of the line is where the field edge was detected. Users can zoom in and determine if this was selected correctly. Additionally, there are two boxes on each of the crosshair axes. The user can examine if these locations were selected correctly. (c) The polar plot users would see for table or collimator walkout. Users can determine that the shape is reasonable and determine where the maximum excursion occurs.

Each of these categories opens a separate window with all tests conducted as listed in Table 1. When the button is pressed for a given test, the processed picture is displayed on the right side of the GUI for the user to assess the quality of the analysis. Additionally, the button turns green or red to indicate if the test passed or failed, based on criteria from MPPG8b.^2^ Examples of images users would see are shown in Figure 3. These images allow the user to confirm the results reported and, if failed, explore why this may have occurred (e.g., a spot obscuring the light field in the relevant area).

The following describes the methodology used for computing each of the tests.

**TABLE 1:** CVQA mechanical tests.

Table Travel & ODI	Field Sizes	Collimator & Table Rotation
Table lateral ± 10 cm Table longitudinal ± 10 cm Table vertical 90 cm, 100 cm, 110 cm SSD 90 cm, 100 cm, 110 cm SSD ODI	5 cm x 5 cm 10 cm x 10 cm 20 cm x 20 cm 40 cm x 40 cm 2 cm over‐travel X‐jaws 10 cm overtravel Y‐jaws	Collimator 90°, 270° Table 90°, 270° Collimator walkout between 90° and 270° Table walkout between 90° and 270°

#### 2.2.1 Table translations

The camera pose (3 DOF Translation and 3 DOF Rotation) calculated from the ArUco markers in the reference image is compared to the pose from the test image. As the camera is stationary, the difference in camera pose provides a measure of the table translation.

#### 2.2.2 ODI

The ODI position is known in relation to the ArUco markers for all three prescribed table heights (90, 100, 110). The actual table height is first determined using the difference in camera pose between the baseline and test images. Based on this height, one of three ROI boxes is placed over the ODI graticule. Gradient detection is performed to determine the pixel locations of the centers of the ODI major tick marks. The crosshair location is determined in a similar way (Figure 3a). A third order polynomial fit is applied to the tick locations to convert the pixel indices to the corresponding ODI scale value. The ODI value is then read out as the point at which the crosshair falls on the ODI scale curve.

#### 2.2.3 Field size

The goal of the field size measurement is to obtain the positions of the four edges of the field, the center of the field, and the crosshair. The image processing is complicated by the presence of the crosshair, which divides the light field into four separate squares. The precise location of the field edge is a sub processing step that uses eight ROI (two on each side, as shown in Figure 3b). These are placed at thirty percent of the field size inward from the field corner, in order to ensure that the ROI are not on top of the ArUco markers for any field size.

The processing steps are as follows:

- Rectify image
  - Correct Image by applying camera matrix and distortion coefficients
  - Flatten the image via perspective transform using the coordinates of the ArUco markers
- Detect field light
  - Remove noise by low‐pass filtering via sequential downward and upward pyramid filters
  - Convert to binary and detect edges via Otsu thresholding
  - Dilate image to blend the four field light quadrants into one field
  - Build a contour map of the image
  - Search the contour map for a square (or octagon in the case of a 40 cm field)
  - If the square has an area of 5 cm x 5 cm or greater, it is flagged as a valid field
- Identify field features
  - ROI boxes are placed 30% inward from the field corners (2 on each side or 8 ROI total)
  - The maximum gradient of each field edge ROI is the precise field edge location
  - The field size is the difference of the maximum gradient points on each side of the field
  - The field center is the midpoint between maximum gradient points
  - The Crosshair location and center are measured in a similar manner to the field size

#### 2.2.4 Collimator/table angle

The tangent is computed between a line drawn from the bottom left corner of the bottom left marker to the bottom right corner of the bottom right marker and the same line in the reference image.

#### 2.2.5 Collimator/table walkout

A video is acquired at a frame rate of 5 fps while the collimator or table is rotated. For each frame, the center of the image is calculated from the intersection of lines drawn from diagonal ArUco markers. The walkout for each frame is determined by the distance from that frame's center to the center of the reference image.

### 2.3 CVQA commissioning

#### 2.3.1 Repeatability

The repeatability was evaluated by setting up the system and repeating each measurement three times without moving anything within the setup over the course of 30 min. This provides a measure of the variability of the system in acquiring new images. The maximum difference between the three repeated measurements was determined.

#### 2.3.2 Reproducibility

The CVQA system was set up on four separate days on the same linac over the course of 24 days. All measurements were recorded, and the maximum difference between different setups was calculated to determine the reproducibility of the system. All measurements were completed by the same two people in order to remove inter‐user variability from this assessment of CVQA.

#### 2.3.3 Accuracy and inter‐user variability

The accuracy of the linac readout was confirmed for table translations using graph paper for lateral and longitudinal translations and three front pointers for vertical translations. The front pointers were verified at linac commissioning by the vendor. Then the CVQA system was set up and 10 cm translations in each direction were tested with 1 mm increments around those translations up to 2 mm as shown in Table 2. The 1 mm incremental translations were determined by the graph paper for lateral and longitudinal translations and the front pointer for vertical translations.

**TABLE 2:** Commissioning test data acquired.

Mechanical Test	Measurement Points
Lateral Table Travel	±9.8, ± 9.9, ± 10.0, ± 10.1, ± 10.2 cm
Longitudinal Table Travel	±9.8, ± 9.9, ± 10.0, ± 10.1, ± 10.2 cm
Vertical Table Travel	0, ± 0.1, ± 0.2, ± 9.8, ± 9.9, ± 10.0, ± 10.1, ± 10.2 cm
Collimator Angle	89°, 89.5°, 90°, 90.5°, 91°, 269°, 269.5°, 270°, 270.5°, 271°
Table Angle	89°, 89.5°, 90°, 90.5°, 91°, 269°, 269.5°, 270°, 270.5°, 271°

Collimator and table walkout were assessed using graph paper. Every 10° between 90° and 270°, the center of the crosshair was marked on the graph paper. CVQA was then run through this same range of angles and the walkout was recorded. Additionally, the table was offset in the longitudinal direction in 1 mm increments and the walkout re‐acquired to determine that the system trended correctly.

Collimator angle was assessed using a digital level with the gantry at 90°. Then the gantry was rotated to 0° and CVQA collimator angles were assessed at 90° ± 1° and 270° ± 1° in 0.5° increments as shown in Table 2. Once collimator angle is verified at a gantry angle of 90° or 270°, no significant collimator rotation should occur without causing one of several motion interlocks, therefore there should be no meaningful difference due to the manual method and CVQA measured at different gantry angles.

Table angle was difficult to assess using the tools available at our institution. Therefore, the linac readout was checked to be accurate at 90° and 270° using graph paper. Then, the system readout was assumed to be accurate for additional angles. The CVQA system was setup and table angles were assessed at 90° ± 1° and 270° ± 1° in 0.5° increments as shown in Table 2.

All photon physicists (8) and residents (4) at our institution were recruited to assess field sizes and ODI. The experience level of physicists ranged from 5 years to over 30 years. The residents included both first‐ and second‐year residents. The CVQA system was set up and then physicists were given graph paper. They were asked to move the jaws to achieve the field sizes listed in Table 1. For each field size, after the physicist had set the jaws, the graph paper was removed and the field size measurement from CVQA was recorded. Physicists were also asked to read the ODI at 90 cm, 100 cm, and 110 cm SSD by first setting up to each position using a front pointer then reading the ODI at that vertical position. A CVQA reading acquired at the same time was compared to the physicists’ ODI readings. The time required to perform the complete set of CVQA tests was measured, as well as the time required for manual performance of the field size tests for each physicist and resident.

### 2.4 Linear accelerator mechanical performance evaluation

CVQA was performed monthly for four years for each of four clinical Varian TrueBeam linear accelerators at Mayo Clinic's Phoenix, Arizona campus. The test was performed primarily by medical physics residents, as well as several clinical physicists. Performance trends of each accelerator were evaluated, and inter‐accelerator performance was examined. Clinical issues and maintenance items identified by CVQA were recorded for future analysis.

## 3 RESULTS

### 3.1 CVQA repeatability and reproducibility

#### 3.1.1 Repeatability

The maximum difference between the three measurements is shown in Table 3. The ODI measurements varied by 0.2 mm or less across the three measurements. The variation of all table translations was within 0.4 mm. All field sizes were within 0.6 mm across the three measurements, and all table rotations were reproducible within 0.03°. Collimator rotations were consistent within 0.04°, collimator walkouts were consistent within 0.6 mm, and table walkouts were consistent within 0.3 mm.

**TABLE 3:** Repeatability and reproducibility of the CVQA system.

Test	Repeatability	Reproducibility	MPPG8.b Action Limit
ODI 90, 100, 110 (mm)	0.2	1	2 mm over clinical range
Table Travel—Vrt, Lng, Lat (mm)	0.4	0.3	Absolute 2 mm Relative 1 mm over 10 cm
Symmetric Field Size X (mm)	0.4	0.5	2 mm per jaw for clinical range of motion
Symmetric Field Size Y (mm)	0.6	0.5	
Asymmetric Field Size X (mm)	0.3	0.4	
Asymmetric Field Size Y (mm)	0.5	0.4	
Over‐travel X (mm)	0.2	0.1	
Over‐travel Y (mm)	0.3	0.3	
Collimator Rotation (^0^)	0.04	0.35	0.5°
Table Rotation (^0^)	0.03	0.3	Absolute 1° Relative 0.5° over 3°
Collimator Walkout (mm)	0.6	0.4	1 mm
Table Walkout (mm)	0.3	0.5	1 mm

#### 3.1.2 Reproducibility

The maximum difference between setups on different days is displayed in Table 3. Overall, all table translations were reproducible within 0.3 mm, all field sizes except 40 cm Y jaws were reproducible within 0.5 mm, and all table rotations were reproducible within 0.5°. The 40 cm Y jaws have a very large penumbra, and the intensity of the light field across this large penumbra was found to vary with time that the light field was on. More detailed information for the repeatability and reproducibility of each individual test is in the supplemental material.

The time for setup and execution of all tests for CVQA was recorded for each reproducibility measurement. The system took on average 5 min to setup and 7 min to complete all mechanical QA tests. For comparison to manual completion of tests, data was recorded for field size measurements when all physicists adjusted jaws to the desired field size. The average time to complete this one test was 29 ± 8 min. This manual time may be longer than other institutions due to the relatively large number of field sizes evaluated.

#### 3.1.3 Accuracy and inter‐user variability

The presented results are relative to the manual measurements that were used as described in 2.3.3. The CVQA recorded table lateral and longitudinal values were accurate within 0.3 mm. The CVQA recorded table vertical values were accurate within 0.9 mm. Vertical values were accurate within 0.1 mm around 100 cm SSD but increased in error with SSD that deviated from 100 cm. This is due to optimizing the camera focus for the 100 cm SSD plane, as this is where most tests are performed. For ODI, the average difference between the physicist reading and CVQA reading was −0.01 ± 0.03 cm, −0.0008 ± 0.07 cm, and 0.03 ± 0.07 cm for 90 cm, 100 cm, and 110 cm SSD, respectively.

**FIGURE 4:**
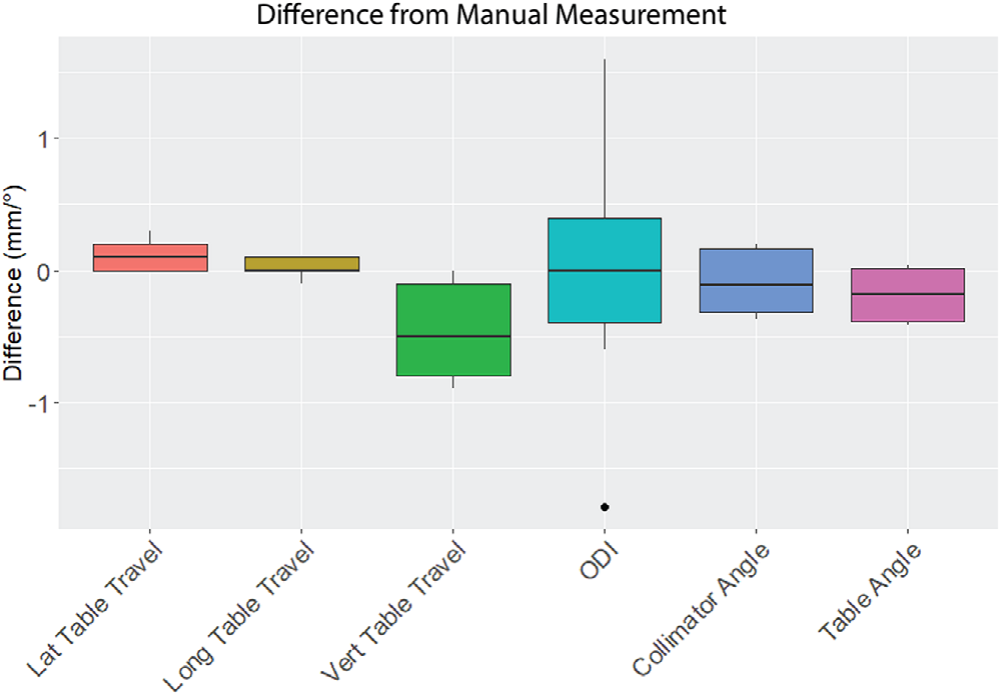
CVQA difference from manual measurements. Boxplots of the difference of the CVQA reading from the manual measurements.

The maximum difference between the digital level reading and the CVQA reading for collimator angles around 90° was −0.37° and 0.2° for angles around 270°. The maximum difference between the linac readout and the CVQA reading for table angles around 90° was −0.41° and 0.02° for angles around 270°. Figure 4 shows a boxplot of the difference from the manual measurements compared to the CVQA reading for table travel, ODI, and collimator/table angle.

The collimator and table walkout from CVQA differed by 0.58 mm and 0.23 mm from the manual method, respectively. The manual methods had many issues which led to a higher value being recorded. First, there is a finite thickness to the crosshairs which makes positioning a pen at the exact center of the intersection difficult. Secondly, the pen has a finite thickness which may overestimate the placement of the point for that angle. And lastly, the pen creates a divot in the center as many angles have the same center. It is difficult to place points on the edge of this divot without overshooting and therefore overestimating the walkout. The 1 mm longitudinal translations showed that the system performed as expected. The 1 mm, 2 mm, and 3 mm translations produced walkouts of 1.42 mm, 2.52 mm, and 3.49 mm, respectively for collimator walkout, and 1.38 mm, 2.36 mm, and 3.46 mm for table walkout. While the manual method has issues, it is an accepted and common method for this measurement and thus utilized here.

**FIGURE 5:**
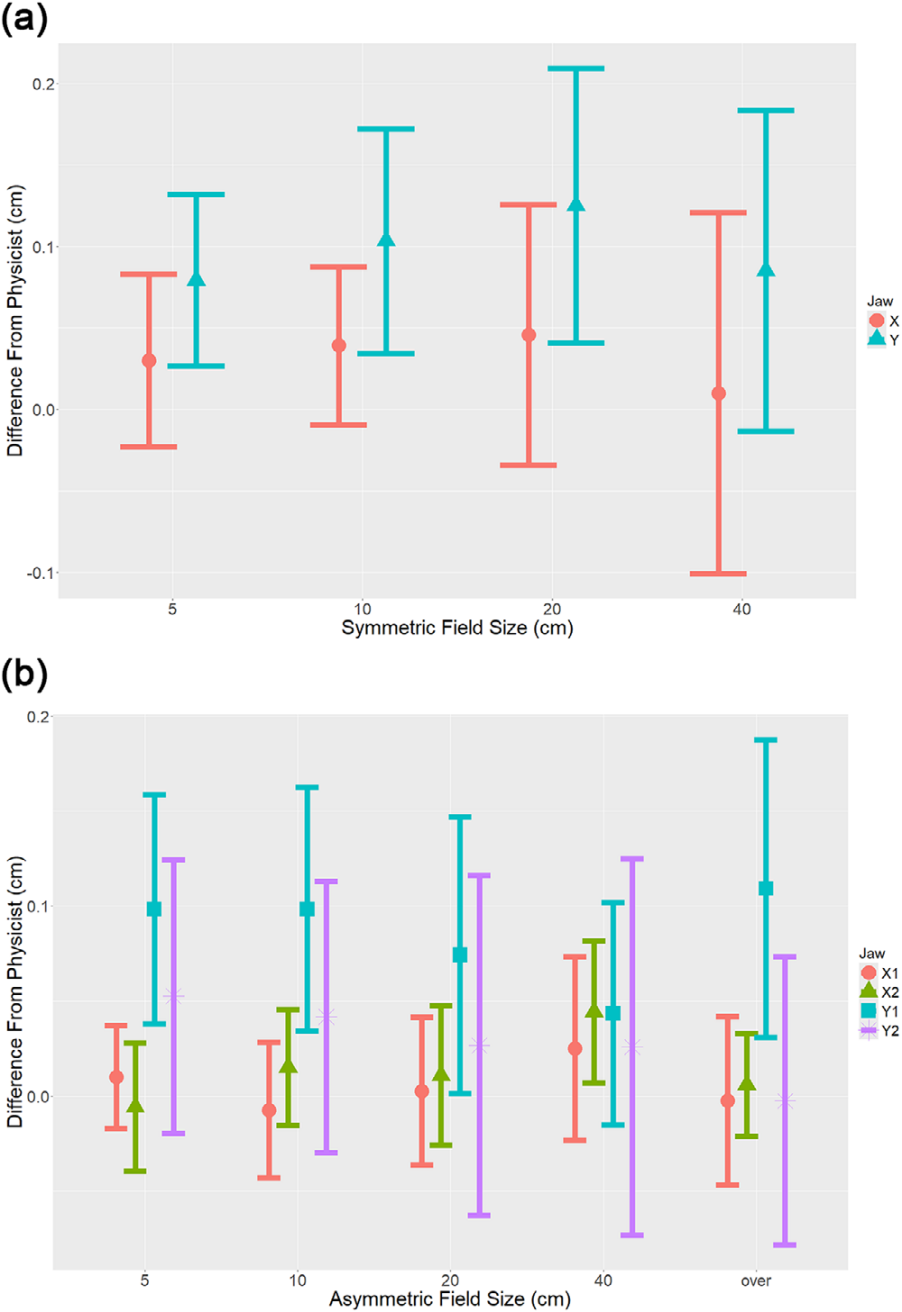
Differences in field size values. Difference between the desired field size and the CVQA reading for the field size set by the physicist for (a) symmetric and (b) asymmetric field sizes. The point location is the average difference and the error bars are the standard deviation across all physicists.

Figure 5 shows the results of the field size assessments of physicists setting jaws and CVQA readings. For the symmetric field sizes, the standard deviation across all physicists was larger than the reproducibility of CVQA for all jaws except for the 40 cm Y jaw. For the asymmetric field sizes, the standard deviation across all physicists was larger than the reproducibility of CVQA for all jaws except for the 40 cm X2 jaw. The standard deviation was larger for the Y jaws than the X jaws for all field sizes and was particularly evident in the asymmetric field sizes where the standard deviation of the Y jaws was about double the standard deviation of the X jaws for each field size. The larger standard deviation in the Y jaws is a result of the less sharp light field edge due to the distance of the jaws from the field light. The increased standard deviation for the Y2 jaws may be due to the greater distance from the camera to the Y2 side of the light field, leading to a small increase in effective pixel size.

### 3.2 Linear accelerator mechanical performance evaluation

CVQA was implemented clinically for monthly mechanical QA in January of 2021 on our four Varian TrueBeam linear accelerators. Two of these machines—TrueBeam D (DTB) and TrueBeam F (FTB) have used CVQA continuously since then, with a few exceptions where monthly mechanical QA was performed manually. TrueBeam C (CTB) and E (ETB) are used for training residents, so CVQA use on them alternates between manual and CVQA for mechanical QA, as residents are trained to do mechanical QA using manual methods before being allowed to use CVQA.

**FIGURE 6:**
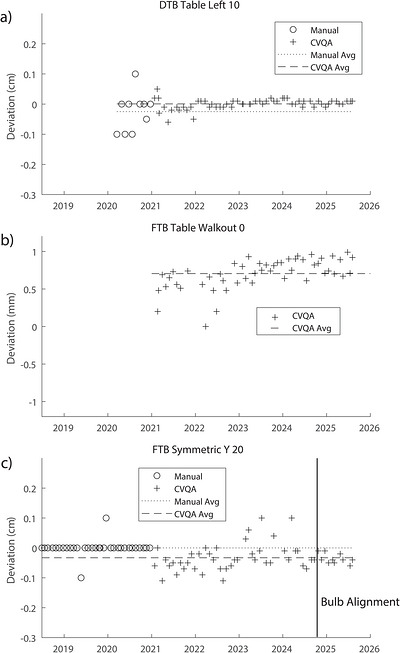
CVQA Findings. (a) CVQA vs manual measurements showing how CVQA captures realistic values which manual measurements are rounded to 0 or increments of 1 mm. The DTB linac did not begin treatment until 2020 so there is no data before that timepoint for that machine. (b) Trends are able to be detected due to the quantitative nature of CVQA with a trend in the table walkout shown. The walkout was originally recorded as pass/fail until CVQA implementation so there is no quantitative data before 2021. (c) Machine issues are able to be detected and resolved as shown where measurements were erratic due to bulb misalignment between the two bulbs in the Truebeam and then variations returned to normal after bulb realignment.

Figure 6a shows the difference between manual and CVQA measurements for lateral table motion. Manual measurements are often exactly at 0 or exactly at 0.1 cm deviation while CVQA shows that the value is not exactly either of these. This is representative of most tests, where the value reported for manual measurements typically shows no deviation or an integer multiple of millimeter or tenths of a degree, and the CVQA results are more continuous, but with a higher level of inter‐measurement noise.

The quantitative and user‐independent nature of CVQA measurements also allows trending of machine performance over time. Figure 6b shows the FTB table walkout over the past 5 years of clinical use. During this time, the walkout has trended upwards by 0.2–0.3 mm. Deviations of this magnitude would be difficult to observe using the manual method of observing the crosshair on graph paper while rotating the table.

CVQA has periodically prompted further investigation which led to service and maintenance interventions. Many other issues found using CVQA are not able to be found with retrospective data review due to the issue being identified and the physicist or resident contacting the engineer to fix. The monthly value was then recorded after the issue had been resolved. One example that is able to be seen in the recorded data is a realignment of the field light bulbs in FTB in October 2024 in response to the collimator rotation being out of tolerance. Prior to the bulb alignment, the field sizes had exhibited some large monthly variations, as seen in Figure 6c between February 2023 and March 2024. This is likely due to the fact that Varian TrueBeam accelerators have two field light bulbs, one of which is randomly selected each time the field light is turned on. Each time the field light is switched on, one of the bulbs is randomly selected, with the primary bulb being selected approximately 70% of the time. If the two field light bulbs are not well aligned with one another, then measurements of field size, collimator walkout, and other tests may show variations from test to test. We suspect that the field size difference measurements exceeding 1 mm in 2023 and 2024 occurred when the second bulb, which was found to be out of alignment, was randomly selected. After bulb alignment in October 2024, this month‐to‐month variability decreased substantially. We did not observe field distortions when we detected misalignment between the two bulbs.

## 4 DISCUSSION

A comparison of mechanical QA results before and after the implementation of CVQA illustrates the differences in measurements from a quantitative light measurement system (CVQA), compared to the more subjective and less precise evaluation possible with the human visual system. Recorded values for field sizes measured with graph paper, for example, tend to change in 1 mm increments. It is difficult for the human visual system to distinguish smaller differences than this in the case of evaluating the penumbra of a field light.^9^, ^10^, ^11^, ^12^

A novel linear accelerator mechanical QA system was created, which includes a camera mount, board, and computer vision analysis tools. Similar to previous work, we were able to automate QA tests.^4^, ^5^, ^6^, ^7^ Unlike several previous works, CVQA did not automate any tests involving the radiation field, as these tests are no longer required for monthly QA by MPPG 8.b or are only executed annually (such as collimator, couch, and gantry radiation isocenter).^2^ In comparison to all other previous work, we were able to design a system that is capable of light field detection for all field sizes. Additionally, while many of the previous studies aimed to demonstrate feasibility, our approach incorporates a robust GUI designed for efficiency and easy integration into the clinic. This has facilitated adoption of CVQA into our monthly practice, which includes a rotation of multiple physicists and residents.

CVQA was shown to be much faster than manual QA testing as it took only a total 12 min for setup and completing all manual QA tests, compared to approximately 30 min to perform only the field size tests. The time for manual performance of other tests was not measured, but the overall time savings is clearly significant. Additionally, the system is precise and accurate as shown through the reproducibility and commissioning measurements. CVQA removes variation from different physicists, which allows for trending data over time and across different machines even with different users performing QA. This is particularly relevant in clinics such as ours, where a large proportion of the monthly linear accelerator QA is performed by trainees. As shown in Table 3 and Figure 6, humans can be more variable than the action limit recommended for mechanical tests by MMPG 8.b,^2^ while CVQA is reproducible and accurate below this action limit. CVQA is a practical tool for use in clinics due to these advantages.

One nuance of the results for manual and CVQA comes from the existence of two independent light bulbs in the Varian TrueBeam light field system. During installation and maintenance, the field light bulbs are adjusted to ensure a consistent field light no matter which bulb is used. However, small differences in the field light position may still occur between bulbs. While these differences are too small to be resolved with the human visual system, they may be more detectable using a precise, quantitative system like CVQA. The selected bulb is not displayed during normal clinical operation, and the bulb selection is not recorded in the treatment record, so it is difficult to assess the impact of field light selection on the consistency of measured results. It is expected that the random selection of the field light bulb increases the variation of CVQA results.

We have noted that CVQA in its current implementation has some sensitivity to the room light level. Consequently, our instructions for use specify a particular combination of room lights for each treatment vault in order to provide maximum reproducibility and accuracy of the test results. Over the course of five years of clinical use, we have found that compliance with these lighting instructions has not been perfect, which has led to a larger degree of uncertainty in the measured results. The mean image intensity is displayed in the GUI. Upper and lower mean image levels for a treatment room should be empirically established during commissioning by evaluating system performance, and those values should be manually entered into the configuration file. A warning message will be displayed if the mean image intensity is outside that range. Additionally, we have found that the ethernet cable connecting the camera to the laptop can provide a small amount of torque on the camera during couch and collimator rotation. If the cable is not managed carefully during these tests, the measured walkout increases. We are refining the light field detection algorithms and developing a wireless camera version of CVQA in order to reduce the impact of user compliance on the test results.

There are several limitations to the current system. Currently, the ODI can only be measured at 90 cm, 100 cm, and 110 cm SSD, due to the need to prepare a unique template image at each SSD to instruct the system where to place boxes for analysis. Future developments would include enhancing this detection to remove the reliance on a template. Additionally, the camera calibration focal length parameter must be manually adjusted to provide accurate couch height measurements. Alternate approaches to improve couch height accuracy are being explored. While we have demonstrated accurate jaw measurements, multi‐leaf collimator‐shaped fields are an area for future work. CVQA has considerable potential for MLC leaf position accuracy and field shape measurements. This capability will be explored in future work. Additionally, the system was designed using an interface mount tray for Varian, therefore, for use on different vendors, different mounts would need to be designed.

## 5 CONCLUSION

CVQA successfully automates mechanical QA tasks, providing an efficient and quantitative tool for monthly linear accelerator testing. It reduces inter‐user variability and enables the trending of linac mechanical performance over time. We have used it for nearly five years in our clinic on four linear accelerators, and it has helped us to identify performance issues and maintenance needs sooner than they would likely have been noticed using standard techniques that rely on the human visual system. It is also preferred by our physicists and residents because of its efficiency and ease of use. CVQA is open source and freely available to academic institutions. Its adoption can improve workflow efficiency and consistency in clinical environments.

## AUTHOR CONTRIBUTIONS

**Rachel B. Ger**: data curation, formal analysis, investigation, methodology, visualization, writing—original draft, writing—review & editing. **Michael D. Armstrong**: data curation, investigation, methodology, resources, software, writing—review & editing. **Daniel G. Robertson**: conceptualization, data curation, formal analysis, investigation, methodology, resources, supervision, visualization, writing—review & editing.

## CONFLICT OF INTEREST STATEMENT

The authors declare no conflicts of interest.

## Supporting information




Supporting Information
