# Supplementary material for: Computer vision mechanical QA: Development, characterization, and five years of clinical performance
Source: J Appl Clin Med Phys. 2026 Jul 15;27(7):e70678. doi: 10.1002/acm2.70678 (PMC13373447; doi:10.1002/acm2.70678)
Supplement: Supplementary file 1 — Supporting Information [file ACM2-27-e70678-s001.docx]

Supplemental Material for “Computer Vision Mechanical QA: Development, Characterization, and Five Years of Clinical Performance”

**Supplemental Table 1. Repeatability and Reproducibility Test Results**

| Test | Repeatability | Reproducibility | MPPG8.b Action Limit |
| --- | --- | --- | --- |
| ODI 90 | 0.2 mm | 1 mm | 2 mm over clinical range |
| ODI 100 | 0 mm | 1 mm |  |
| ODI 110 | 0.2 mm | 1 mm |  |
| Vrt -10 | 0.1 mm | 0.1 mm | Absolute 2 mm, Relative 1 mm over 10 cm |
| Vrt +10 | 0.2 mm | 0.1 mm |  |
| Lng +10 | 0 mm | 0.3 mm |  |
| Lng -10 | 0.1 mm | 0.1 mm |  |
| Lat +10 | 0 mm | 0.1 mm |  |
| Lat -10 | 0.4 mm | 0.1 mm |  |
| Symmetric 40 X | 0.4 mm | 0.3 mm | 2 mm per jaw for clinical range of motion |
| Symmetric 40 Y | 0.3 mm | 0.3 mm |  |
| Symmetric 20 X | 0.1 mm | 0.5 mm |  |
| Symmetric 20 Y | 0.6 mm | 0.2 mm |  |
| Symmetric 10 X | 0.1 mm | 0.1 mm |  |
| Symmetric 10 Y | 0.3 mm | 0.5 mm |  |
| Symmetric 5 X | 0 mm | 0.3 mm |  |
| Symmetric 5 Y | 0.2 mm | 0.3 mm |  |
| Asymmetric 40 X1 | 0.3 mm | 0.3 mm |  |
| Asymmetric 40X2 | 0.2 mm | 0.4 mm |  |
| Asymmetric 40 Y1 | 0.3 mm | 0.2 mm |  |
| Asymmetric 40Y2 | 0.5 mm | 0.4 mm |  |
| Asymmetric 20 X1 | 0 mm | 0.1 mm |  |
| Asymmetric 20X2 | 0.1 mm | 0.1 mm |  |
| Asymmetric 20 Y1 | 0.1 mm | 0.2 mm |  |
| Asymmetric 20 Y2 | 0.3 mm | 0.3 mm |  |
| Asymmetric 10 X1 | 0.1 mm | 0.2 mm |  |
| Asymmetric 10 X2 | 0.3 mm | 0.2 mm |  |
| Asymmetric 10 Y1 | 0.2 mm | 0.2 mm |  |
| Asymmetric 10 Y2 | 0 mm | 0.4 mm |  |
| Asymmetric 5 X1 | 0 mm | 0.1 mm |  |
| Asymmetric 5 X2 | 0 mm | 0.2 mm |  |
| Asymmetric 5 Y1 | 0 mm | 0.2 mm |  |
| Asymmetric 5 Y2 | 0.1 mm | 0.4 mm |  |
| Over-travel X1 | 0 mm | 0.1 mm |  |
| Over-travel X2 | 0.2 mm | 0.1 mm |  |
| Over-travel Y1 | 0 mm | 0.2 mm |  |
| Over-travel Y2 | 0.3 mm | 0.3 mm |  |
| Collimator 90⁰ | 0.04° | 0.28° | 0.5° |
| Collimator 270⁰ | 0.02° | 0.35° |  |
| Collimator Walkout | 0.6 mm | 0.38 mm | 1 mm |
| Table 90⁰ | 0.03° | 0.3° | Absolute 1°, Relative 0.5° over 3° |
| Table 270⁰ | 0.02° | 0.48° |  |
| Table Walkout | 0.3 mm | 0.45 mm | 1 mm |


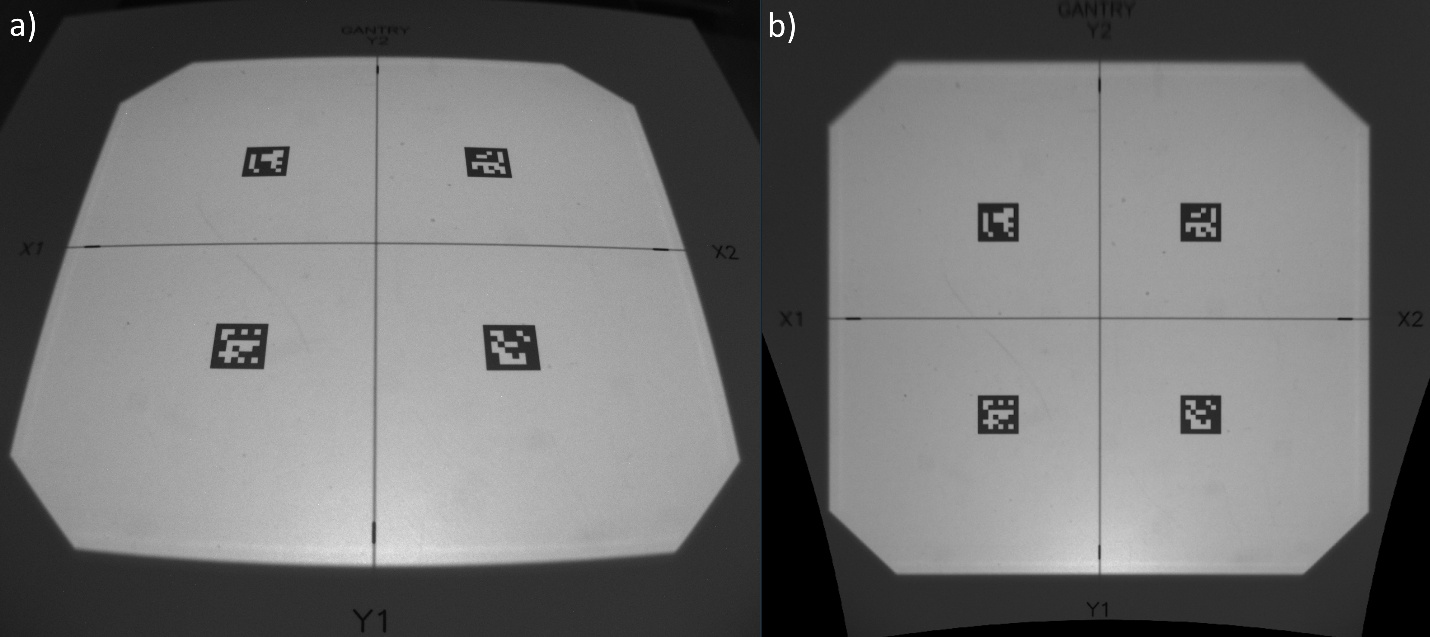


**Supplemental Figure 1. CVQA Camera Images.** (a) The unflattened view from the camera and (b) the flattened view from the camera used for field size detection, collimator/table rotation, collimator/table walkout, and ODI.
